# The influence of TCM constitutions and neurocognitive function in elderly Macau individuals

**DOI:** 10.1186/s13020-021-00441-2

**Published:** 2021-04-13

**Authors:** Zhuo Zhang, Yaochen Chuang, Xinyu Ke, Ji Wang, Youhua Xu, Yonghua Zhao, Ying Bian

**Affiliations:** 1 State Key Laboratory of Quality Research in Chinese Medicine, Institute of Chinese Medical Sciences, University of Macau, Taipa, Macao China; 2 Faculty of Chinese Medicine, Macau University of Science and Technology, Macao, China; 3 Kiang Wu Nursing College of Macau, Macao, China; 4 Traditional Chinese Medicine Constitution Research Center, Beijing University of Chinese Medicine, Beijing, China

**Keywords:** Elderly Macau individuals, Neurocognitive function, Traditional Chinese medicine constitution

## Abstract

**Background:**

Traditional Chinese medicine (TCM) constitution contributes to predicating disease occurrence and pathological progress. In this study, we investigate the correlation between TCM constitution and neurocognitive function in elderly Macau individuals.

**Methods:**

A total of 313 older adults from elderly healthcare centers were recruited at random. The data of gender, age, education, sleeping hours, physical activities were collected, and the Geriatric Depression Scale, Hong Kong version of the Montreal Cognitive Assessment (MoCA) and categories of TCM constitution were administered.

**Results:**

Of the 313 elderly individuals enrolled in this study, 86 (27.48%) were of balanced constitution. Among the other categories of TCM constitution, the most was Yin-deficiency (23.32%), followed by 53 (16.93%) with Phlegm-dampness. The average neurocognitive score of all elderly individuals was 18.01 ± 6.25. After adjusting for all possible confounds, multiple linear regression analysis showed that Qi-depressed constitution and neurocognitive scores were negatively correlated (β = − 2.66, 95%CI − 4.99 ~ − 0.33), Meanwhile, Yin-deficient constitution and neurocognitive scores were negatively correlated (β = − 2.10, 95%CI − 3.73 ~ − 0.47). Compared with balanced constitution, Qi-depressed constitution mainly affected visual–spatial ability dimension (β = − 0.91, 95%CI − 1.54 ~ − 0.28) and naming dimension (β = − 0.64, 95%CI − 1.04 ~ − 0.25), Yin-deficient constitution mainly affected visual space dimension (β = − 0.53, 95%CI − 0.97 ~ − 0.08).

**Conclusion:**

Qi-depressed and Yin-deficient constitutions are associated with and contributed to the decline of neurocognitive function in senior adults, especially visual–spatial ability and naming dimensions. Further investigations into how TCM constitutions interact with neurocognitive function are needed.

## Introduction

With the number of populations aged 65 years or over increases rapidly, China is facing a serious increasing burden of neurocognitive disorders (NCDs). Cognitive impairment is commonly underestimated, and a recent mate-analysis had shown that the prevalence of NCDs in elderly Chinese people was 15.4% [1]. Moreover, it is estimated by 2050, the number of elderly people will double to 1.5 billion [2]. Therefore, early screening of neurocognitive function in elderly individuals is of vital importance.

The Montreal Cognitive Assessment (MoCA) is a brief screening tool for assessment of neurocognitive impairment from various domains. The MoCA is a one-page test with a maximum of 30 points and developed and validated by Nasreddine et al. [3]. It usually can be completed within 15 min, so has been translated and adopted in many countries. Our study employ the Hong Kong version of MoCA (HK-MoCA) which is conducted in Cantonese [4]. The MoCA assesses cognitive domains with 10 items including tests on visual–spatial ability, naming, attention, language, abstract reasoning, memory, orientation to time and place. A score under 21 indicates the possibility of neurocognitive impairment. The MoCA is validated to have good sensitivity and specificity, and the reliability of HK-MoCA has also been validated under various clinical conditions including mild neurocognitive disorder, major neurocognitive disorder, dementia, Alzheimer disease, stroke, cerebral small vessel disease etc [4–7].

Since proposed, the constitutional theory of Chinese medicine has become the reference standard for constitutional researchers and is widely applied in clinical practice and disease control. In the theoretical system of Traditional Chinese Medicine (TCM), one’s constitution refers to personal features adapted to natural and social environment and shows one’s susceptibilities to diseases and responses to outside stimuli [8–10]. TCM constitution is divided into nine types proposed by Wang Qi [11]. The category of TCM constitution provides the basis of individualized treatments and achieving optimal therapeutic effects for clinicians, which contributes to curing diseases or preventing diseases from bad prognosis [9, 10, 12].

Some studies have revealed the relation between TCM constitutions and neurocognitive function [13–16]. However, rare studies focused on Chinese elderly population, and the conclusions are still inconsistent. Therefore, this study investigates the characteristics of TCM constitution and evaluates the correlation between TCM constitution and neurocognitive function in elderly individuals of Macau community. In addition, we investigate the correlation of TCM constitution categories with neurocognitive function dimensions, which may shed new light on intervening NCDs in early status from the approach of susceptible TCM constitutions, which contributes to adopting more effective and precise therapy.

## Methods

### Study design and subjects

The study protocol has been approval by ethics committee of University of Macau. Based on the administrative structure, a stratified random sampling method was used to acquire the represented sample. Firstly, the Macau was divided into three section (Peninsula, Taipa and Coloane), 1 elderly healthcare center was randomly selected from each administrative district. Areia Preta Minghui Nursing Home, PouTai Elderly Service Integrated Center, Enhui Elderly Service Integrated Center from Peninsula, Taipa and Coloane, respectively, were finally selected in this survey. Secondly, 400 participants were randomly selected from the elderly registered in the three elderly healthcare centers from September to December 2019. With incomplete information (n = 56) and mixed constitution (n = 31) were excluded, a total of 313 elderly individuals were finally included in the present research. Criteria for selection of research subjects: (1) Chinese residents in Macau; (2) 65 years of age or above; (3) No intellectual and language communication barriers, able to understand and answer the questions in Cantonese; (4) No major diseases in the past year. Participants who suffered from tumor, heart failure, mental illness and other serious systemic diseases and could not complete the questionnaire even with assistance were excluded. The investigators were college students or graduate students with a background in Chinese medicine or medical care, and had fluent Cantonese communication skills. All investigators had undergone uniform training. Before the survey, the investigators explained the significance, purpose, time requirements and research ethics principles to each participant, and a written informed consent form was obtained. Then investigators individually interviewed each participant, read out each question clearly in the questionnaires for participants to answer, and filled in their answers in the questionnaires.

We had calculated the sample size by the following formula before investigation.

$$\mathrm{N}=\frac{{z}^{2}p(1-p)}{{e}^{2}}$$

With a maximum response distribution rate (p = 0.5, e = 0.1p) and 90% confidence level (z = 1.64),

269 samples were needed in this study, and taking into account the non-response rate, we expanded the sample size to 400. This study finally included 313 subjects, which met the sample size.

### Study variables

In this study, neurocognitive function was used as the dependent variable, and TCM constitutions were used as the independent variable. The neurocognitive function of participants was evaluated by the Montreal Cognitive Assessment-Hong Kong Version [4]. The MoCA is a psychologically effective and reliable tool. The scale includes cognitive dimensions of visual–spatial ability, naming, attention, language, abstract reasoning, memory, orientation to time and place. The scale consists of 30 items. The higher the score, the better the neurocognitive function. Using Wang Qi’s Nine TCM Constitution questionnaire for the elderly [18] to investigate TCM constitutions, there are 9 physical fitness subscales including balanced constitution, Qi-deficient, Qi-depressed, Dampness-heat, Phlegm-dampness, Blood-stasis, Yin-deficient, Yang-deficient, and special constitutions with a total of 33 questions. Questions were scored according to 5 levels. The original score and conversion score were calculated, and then related TCM constitution was determined according to the calculated score.

### Covariates

The covariates adjusted in this study included gender (male, female), age (< 75 years, ≥ 75 years), education level (illiteracy, primary schools and below, middle school and above), sleep time (< 7 h, 7–9 h, ≥ 9 h), regular exercise (no, yes), depression (no, yes). The age was divided based on the WHO classification criteria for the elderly. The sleep time stratification was based on the joint consensus statement of the American academy of sleep medicine and sleep research society on the recommended amount of sleep [17]. The Geriatric Depression Scale (GDS-15) was used to evaluate mental health of participants. There are 15 items in the scale, which are answered with "yes" or "no". The total is 15 points, and the higher the score, the more obvious the depressive symptoms. The total score greater than 5 indicates the possibility of depression.

### Statistical analysis

Excel was used to establish a database, and all data was double entered to ensure accurate entry. The basic characteristics of the research objects were described by counts and proportions, neurocognitive scores were described by mean ± SD, and Student t test or variance analysis was used for comparison between different groups. Multiple linear regression was employed to analyze the relationship between TCM constitutions and neurocognitive functions, using neurocognitive scores as dependent variable and TCM constitutions as independent variable. Three models were established. Model 1 adjusted for nothing. Model 2 adjusted for gender and age. Model 3 adjusted for the variables in the model 2 and education, sleeping hours, exercise regularly, depression. In order to analyze the impact of Qi-depressed and Yin-deficient constitutions on neurocognitive function, the scores of dimensions of visual–spatial ability, naming, attention, language, abstract reasoning, memory and orientation to time and place were collected as dependent variables, respectively, and TCM constitutions were used as independent variables. After controlling gender, age, regular exercise, depression, education level, and sleeping time, multiple linear regression models were established.

The statistical analysis in the study was completed with stata16.0, and P < 0.05 was regarded as statistical significance.

## Results

### Background information of subjects

400 elderly individuals were invited and all of them filled out the questionnaire, with a response rate of 100%. 313 participants were included in the study after excluding those with severe lack of information and mixed constitution. Of them, the average age was 77.10 ± 8.23 years old. Women accounted for the majority (82.43%). The education level of most participants was concentrated in middle school and below (55.59%). More than half of them slept ≥ 7 h, 76.36% of participants exercised regularly, and 24.28% suffered from depression. Among all participants, 86 individuals (27.48%) had a balanced constitution. Among other types of constitution, the most were individuals with Yin-deficiency (23.32%), followed by individuals with Phlegm-dampness (16.93%). The average neurocognitive score of all participants was 18.01 ± 6.25. There were differences in neurocognitive scores between different age groups, education levels, exercise status, depression status, and TCM constitution categories (Table 1).

**Table 1:** Sociodemographic characteristics and neurocognitive scores of the study subjects

	N (%)	Neurocognitive scores ^#^
Gender		
Male	55 (17.57)	18.42 ± 6.21
Female	258 (82.43)	17.93 ± 6.27
Age		
≤ 75	126 (40.26)	20.44 ± 4.98**
≥ 75	187 (59.74)	16.37 ± 6.49
Education		
Illiteracy	77 (24.60)	12.70 ± 5.36**
Primary schools and below	174 (55.59)	19.04 ± 5.57
Middle school and above	62 (19.81)	21.73 ± 4.85
Sleeping hours		
< 7	74 (23.64)	18.65 ± 5.77
7–9	139 (44.41)	19.17 ± 6.00
≥ 9	100 (31.95)	15.94 ± 6.48
Exercise regularly		
No	74 (23.64)	16.72 ± 7.03*
Yes	239 (76.36)	18.41 ± 5.95
Depression		
No	237 (75.72)	18.52 ± 6.12
Yes	76 (24.28)	16.42 ± 6.42*
TCM constitutions		
Balance	86 (27.48)	19.90 ± 5.98*
Qi-deficiency	17 (5.43)	17.94 ± 6.75
Qi-depression	28 (8.95)	15.36 ± 6.74
Dampness-heat	2 (0.64)	15.00 ± 9.90
Special constitution	7 (2.24)	18.43 ± 6.85
Phlegm-dampness	53 (16.93)	18.21 ± 5.83
Blood-stasis	12 (3.83)	17.75 ± 6.20
Yin-deficiency	73 (23.32)	16.77 ± 6.09
Yang-deficiency	35 (11.18)	18.03 ± 6.24

^*^P < 0.05 **P < 0.01

^#^ Reported as $\stackrel{-}{\mathrm{x}}$±SD

### The correlation of TCM constitution category with neurocognitive scores

Multiple linear regression analysis showed that after adjusting for all possible confounds, Qi-depressed constitution and neurocognitive function were negatively correlated (β = − 2.66, 95% CI − 4.99 ~ − 0.33), Meanwhile, Yin-deficient constitution and neurocognitive function were negatively correlated (β = − 2.10, 95% CI − 3.73 ~ − 0.47). There was no statistical difference in the correlation of other TCM constitution categories with neurocognitive function (Table 2).

**Table 2:** The correlation of TCM constitution categories with neurocognitive scores

Category of TCM constitution	Model 1		Model 2		Model 3	
	β (95%CI)	P	β (95%CI)	P	β (95%CI)	P
Qi-deficiency	− 1.95 (− 5.17 ~ 1.27)	0.233	− 1.18 (− 4.26 ~ 1.89)	0.450	− 1.10(− 3.78 ~ 1.58)	0.421
Qi-depression	− 4.54 (− 7.18 ~ − 1.90)	0.001	− 4.26 (− 6.77 ~ − 1.75)	< 0.001	− 2.66 (− 4.99 ~ − 0.33)	0.025
Dampness-heat	− 4.90 (− 13.57 ~ 3.78)	0.268	− 2.79 (− 11.09 ~ 5.50)	0.508	− 2.37 (− 9.63 ~ 4.89)	0.520
Special constitution	− 1.47 (− 6.24 ~ 3.30)	0.545	− 1.74 (− 6.28 ~ 2.80)	0.452	− 2.21 (− 6.21 ~ 1.78)	0.276
Phlegm-dampness	− 1.69 (− 3.81 ~ 0.43)	0.118	− 1.08 (− 3.10 ~ 0.95)	0.296	− 0.67 (− 2.44 ~ 1.10)	0.458
Blood-stasis	− 2.15 (− 5.88 ~ 1.59)	0.260	− 1.70 (− 5.29 ~ 1.88)	0.350	− 2.45(− 5.59 ~ 0.70)	0.127
Yin-deficiency	− 3.13 (− 5.06 ~ − 1.20)	0.002	− 2.66 (− 4.51 ~ − 0.82)	0.005	− 2.10 (− 3.73 ~ − 0.47)	0.012
Yang-deficiency	− 1.87 (− 4.30 ~ 0.57)	0.132	− 1.17 (− 3.51 ~ 1.17)	0.327	− 0.62 (− 2.66 ~ 1.42)	0.552

Model 1 adjusted for nothing. Model 2 adjusted for gender and age. Model 3 adjusted for the variables in Model 2 and education, sleeping hours, exercise regularly, depression. The reference group was balanced constitution. The variance inflation (VIF) of each variable in Model 3 ranged from 1.04 to 1.73, and the mean VIF was 1.33

### Scores of neurocognitive dimensions in different TCM constitution categories

The average scores of visual–spatial ability, naming, attention, language, abstract reasoning, memory, and orientation to time and place of all participants were 2.06 ± 1.56, 2.06 ± 0.93, 4.09 ± 1.72, 2.51 ± 0.73, 0.73 ± 0.78, 1.48 ± 1.72, 5.08 ± 1.42, respectively. Scores of various dimensions of TCM constitution categories were presented in Table 3. Visual–spatial ability and naming scores were statistically different among different categories of TCM constitution (P < 0.05) (Table 3).

**Table 3:** Scores of neurocognitive dimensions in different categories of TCM constitution

Category of TCM constitution	Visual–spatial ability	Naming	Attention	Language	Abstract reasoning	Memory	Orientation to time and place
Balance	2.50 ± 1.55*	2.27 ± 0.86*	4.59 ± 1.47	2.64 ± 0.65	0.85 ± 0.80	1.77 ± 1.85	5.28 ± 1.37
Qi-deficiency	2.18 ± 1.78	2.24 ± 0.83	4.00 ± 1.94	2.47 ± 0.72	0.76 ± 0.75	1.12 ± 1.32	5.18 ± 1.55
Qi-depression	1.29 ± 1.46	1.50 ± 1.17	3.57 ± 1.83	2.39 ± 0.57	0.61 ± 0.79	1.21 ± 1.62	4.79 ± 1.71
Dampness-heat	1.50 ± 2.12	2.50 ± 0.71	3.00 ± 2.83	2.50 ± 0.71	0 ± 0	1.50 ± 2.12	4.00 ± 2.83
Special constitution	2.29 ± 1.80	2.29 ± 0.76	4.14 ± 2.34	2.43 ± 0.79	0.86 ± 0.9	1.43 ± 1.40	5.00 ± 1.00
Phlegm-dampness	2.11 ± 1.25	2.21 ± 0.84	3.89 ± 1.91	2.47 ± 0.85	0.70 ± 0.80	1.60 ± 1.75	5.23 ± 1.28
Blood-stasis	2.42 ± 1.73	2.00 ± 0.60	4.33 ± 1.87	2.33 ± 0.98	0.33 ± 0.78	1.33 ± 1.61	5.00 ± 1.04
Yin-deficiency	1.79 ± 1.63	1.93 ± 0.90	3.90 ± 1.63	2.41 ± 0.80	0.73 ± 0.73	1.16 ± 1.65	4.84 ± 1.45
Yang-deficiency	1.89 ± 1.51	1.94 ± 1.08	3.94 ± 1.66	2.69 ± 0.53	0.71 ± 0.83	1.69 ± 1.79	5.17 ± 1.50

^*^ P < 0.05

### The correlation of Qi-depressed and Yin-deficient constitutions with neurocognitive dimensions

After controlling for the covariates, multiple linear regression showed that individuals with Qi-depressed constitution had lower scores on visual–spatial ability (β = -0.91, 95% CI − 1.54 ~ − 0.28) and naming (β − 0.64, 95% CI − 1.04 ~ − 0.25) compared with Balanced constitution, and individuals with Yin-deficient constitution had lower scores on visual–spatial ability (β = − 0.53, 95% CI − 0.97 ~ − 0.08) (Table 4).

**Table 4:** The correlation of Qi-depressed and Yin-deficient constitutions with neurocognitive dimensions

	Qi-depression		Yin-deficiency	
	Β (95%CI)	P	β (95%CI)	P
Visual–spatial ability	− 0.91 (− 1.54 ~ − 0.28)	0.005	− 0.53 (− 0.97 ~ − 0.08)	0.020
Naming	− 0.64 (− 1.04 ~ − 0.25)	0.001	− 0.24 (− 0.52 ~ 0.03)	0.086
Attention	− 0.55 (− 1.22 ~ 0.11)	0.103	− 0.39 (− 0.85 ~ 0.08)	0.102
Language	− 0.07 (− 0.37 ~ 0.24)	0.657	− 0.16 (− 0.37 ~ 0.06)	0.150
Abstract reasoning	− 0.17 (− 0.52 ~ 0.18)	0.332	− 0.08 (− 0.32 ~ 0.17)	0.528
Memory	− 0.21 (− 0.96 ~ 0.54)	0.580	− 0.48 (− 1.00 ~ 0.05)	0.074
Orientation to time and place	− 0.11 (− 0.70 ~ 0.49)	0.727	− 0.23 (− 0.65 ~ 0.18)	0.269

Age, education, exercise regularly, depression, sleeping hours were adjusted in the models

## Discussion

With the increase of age, brain volume of the elderly begins to shrink, so neurocognitive functions are gradually deteriorated. Neurocognitive impairment is an important clinical feature in the early stage of dementia [18]. An older person with neurocognitive impairment will not only reduce his healthy life expectancy, but also have a significant impact on mental health and quality of life of his family caregivers [19]. Therefore, by exploring relevant factors that affect neurocognitive function, it is possible to detect neurocognitive impairment early and find effective interventions to improve the quality of life of the elderly.

Scholars have been trying to observe human health from a holistic and dynamic perspective, interpreting individual differences through the classification of physical fitness, and trying to define and classify the laws between individual differences and diseases from different perspectives to guide disease prevention and clinical practice [11, 20–23]. Wang Qi’s nine types of TCM constitution has been widely applied in Chinese medicine and considered the reference standard for constitutional researchers. From the perspective of TCM constitution theory, balanced constitution represents the overall state of health, and people with biased constitution are prone to certain diseases [24]. With the deficiency of Yin, Yang, Qi and blood of the elderly, the physiological functions are declining [25]. Geriatric diseases are gradually increasing, and there are relatively fewer balanced constitutions and more biased constitutions. Therefore, this study enlightened to establish comprehensive individualized health model and contributed to precision medicine.

The correlation between TCM constitutions and neurocognitive function is currently unclear. Yin of the elderly in five zang-organs has been deficient physiologically flowed by ageing, especially kidney Yin. Kidney, being innate foundation, is responsible for storing essence, and essence is predominant origin of brain marrow governing higher nervous activity. To put it simply, deficiency of Yin is lack of tangible substances such as blood, essence and body fluid, which leads to relative prosperity of Yang on the opposite side [26, 27]. Due to the decline of Yin essence in kidney, the constitution of the elderly is also biased towards Yin deficiency. This study found that Yin deficient constitution was negatively correlated to neurocognitive scores (β = − 2.10, 95% CI − 3.73 ~ − 0.47). This is consistent with Zhang YP’s survey conducted among residents over 50 years of age in Jinan City [16] and Liu ZZ’s study conducted in Fuzhou City [28], and inconsistent with the findings of Sun et al. [15] and Zeng et al. [14]. It can be explained that the formation of biased TCM constitution is perhaps influenced by complicated factors, e.g., education, genetic, acquired living habits and different pathological states so on. Therefore, the study of correlation of biased TCM constitutions with NCDs should focus on population in specific region, which tries to ensure the consistency of intervened factors. Additionally, our research further demonstrated the main correlation between Yin deficient constitution and visual–spatial ability (β = − 0.53, 95% CI  − 0.97 ~ − 0.08) in NCDs. These results suggest that syndrome differentiation and treatment of TCM should invigorate kidney Yin, which contributes to complementing brain marrow and nourishing the mind against NCDs in Macau elderly individuals.

Consistent with the survey conducted among the elderly in Fuzhou [28], our study also found that Qi-depressed constitution was negatively correlated with neurocognitive function ((β = − 2.66, 95% CI − 4.99 ~ − 0.33), compared with balanced constitution. According to the theory of TCM visceral manifestation in the "Huang Di Nei Jing"[29], liver governs free flow of Qi and design of strategy, which exerts central actions on regulating emotion and mental activity. If the elderly have depressed emotion, which causes liver Qi stagnation, they will lose their judged ability for identifying exogenous objects, and their comprehension and memory will decline. If the status lasts long time, liver Qi will be weaken, deficiency of Qi does not hide the will, and is prone to NCDs due to lack of energy and forgetfulness [29]. This study also found that Qi-depressed constitution had close relation with the dimensions of visual–spatial ability (β = − 0.91, 95% CI − 1.54 ~ − 0.28) and naming (β = − 0.64, 95% CI − 1.04 ~ − 0.25), suggesting liver Qi depression played predominant role in the pathogenesis of NCDs. Therefore, syndrome differentiation and treatment of TCM should focus on soothe liver and relieve depression for intervening NCDs in Macau.

**Fig. 1:**
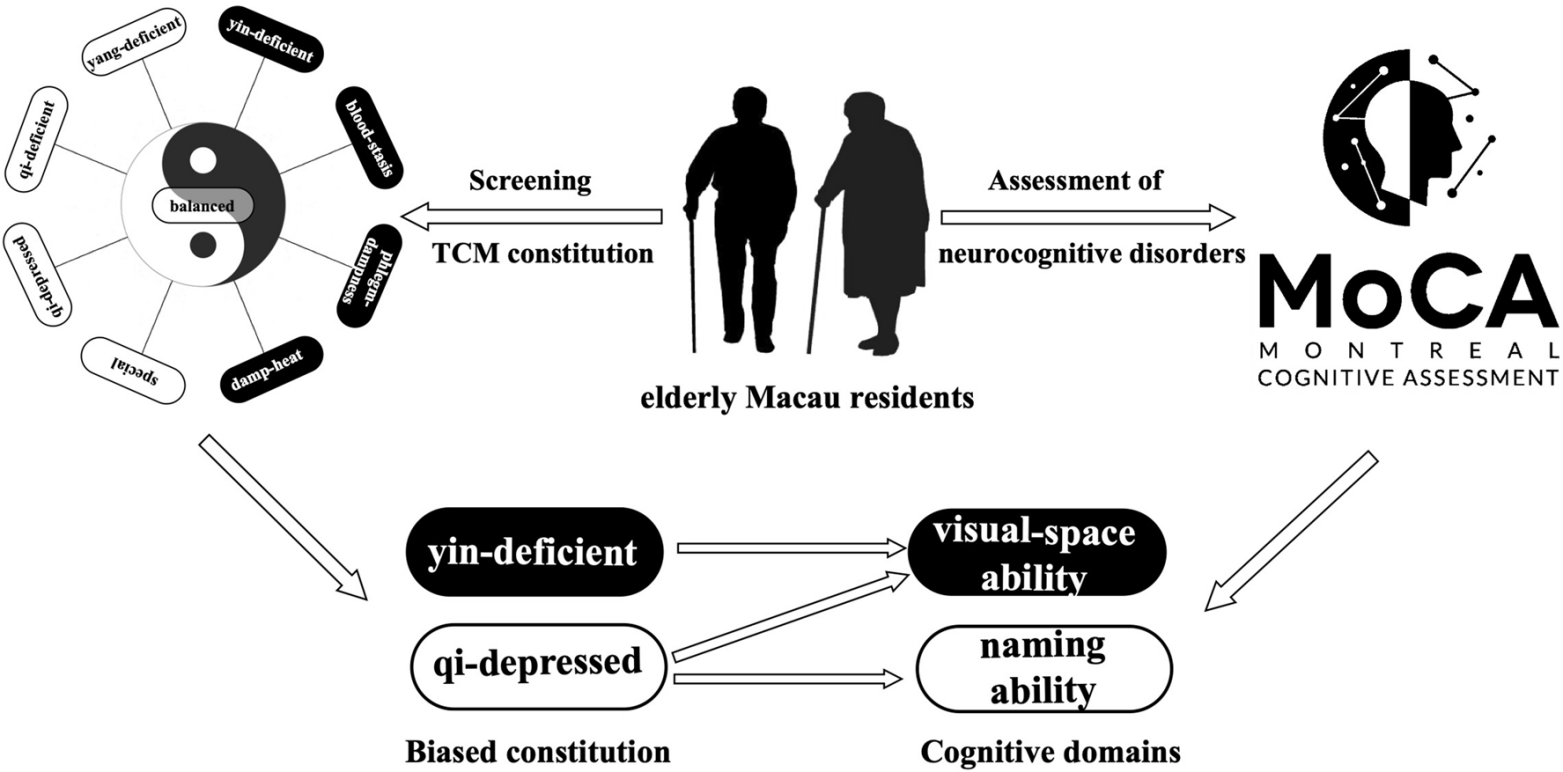
Association between TCM constitutions and neurocognitive functions among elderly Macau individuals

To our knowledge, this is the first study on the correlation between TCM constitutions and neurocognitive functions in elderly Macau individuals (Fig. 1). Although a series of important research have been made in the fields of neuropathology, molecular genetics and other related NCDs in recent years, there is still no cure for NCDs, and only symptomatic treatment is administered for improvement of some symptoms [30, 31]. Therefore, early diagnosis and intervention are extremely important. The purpose of our survey is to assess the correlation between TCM constitutions and neurocognitive function in the elderly of Macau. At the same time, it explains the risk of neurocognitive impairment from the perspective of unique TCM constitution and provides a basis for prevention and treatment of neurocognitive impairment. Our study helps policy makers to realize that TCM constitutions affect the susceptibility and symptomatic tendency of diseases and provides new ideas for prevention and treatment of neurocognitive impairment based on the characteristics of Yin-deficient and Qi-depressed constitutions.

There are some limitations in this study. Based on a cross-sectional study on the relationship between TCM constitutions and neurocognitive functions, it cannot determine the direction of the relationship between TCM constitutions and neurocognitive functions. It is only a preliminary exploration of the correlation between TCM constitution and cognitive function in the elderly in Macao, so it should be interpreted with cautiously in clinical practice. Well-designed prospective experiments were further required to verify the relationship. Although the study considers the effects of gender, age, education level, sleep time, regular exercise, and depression, it cannot consider all variables related to cognition of the elderly in Macau. Furthermore, compared with the elderly in the community from other places, they may have higher health awareness and social support, so their neurocognitive function scores may be better, which affects the extrapolation of research conclusions. Follow-up research should reasonably increase the sample content and conduct multi-center and large sample surveys to provide reliable evidence for the basic research of TCM syndrome epidemiology.

## Conclusion

Our results demonstrate that there is the significant correlation between Qi-depressed and Yin-deficient constitutions and neurocognitive functions among elderly individuals in Macau, suggesting characteristics of TCM constitution might influence pathophysiology of NCDs, especially Qi-depressed constitution having close relationship with the dimensions of visual–spatial ability and naming, and Yin-deficient constitution mainly affecting visual space dimension. Therefore, targeting the two TCM constitutions, interventions performed as early as possible is beneficial for prevention and attenuation of NCDs pathological progress in elderly Macau individuals.

## Data Availability

The data used and/or analyzed during the study are available from the corresponding author on reasonable request.

## Abbreviations

TCM constitution: Traditional Chinese medicine constitution
NCDs: Neurocognitive disorders
HK-MoCA: The Hong Kong version of Montreal Cognitive Assessment
GDS-15: The Geriatric Depression Scale

## References

[1] Deng Y, Zhao S, Cheng G, Yang J, Li B, Xu K, Xiao P, Li W, Rong S. The prevalence of mild cognitive impairment among Chinese people: a meta-analysis. Neuroepidemiology 2021:1–13. 10.1159/000512597 33756479

[2] Zueva AS, Khrolenko TS (2019). Population ageing: demographic security threat or silver industry development potential. J Public Admin.

[3] Nasreddine ZS, Phillips NA, Bédirian V, Charbonneau S, Whitehead V, Collin I, Cummings JL, Chertkow H (2005). The Montreal Cognitive Assessment, MoCA: a brief screening tool for mild cognitive impairment. J Am Geriatr Soc.

[4] Wong A, Xiong YY, Kwan PW, Chan AY, Lam WW, Wang K, Chu WC, Nyenhuis DL, Nasreddine Z, Wong LK (2009). The validity, reliability and clinical utility of the Hong Kong Montreal Cognitive Assessment (HK-MoCA) in patients with cerebral small vessel disease. Dement Geriatr Cogn Disord.

[5] Pan IMY, Lau MS, Mak SC, Hariman KW, Hon SKH, Ching WK, Cheng KM, Chan CF (2020). Staging of dementia severity with the Hong Kong version of the montreal cognitive assessment (HK-MoCA)'s. Alzheimer Dis Assoc Disord.

[6] Yeung PY, Wong LL, Chan CC, Leung JL, Yung CY (2014). A validation study of the Hong Kong version of Montreal Cognitive Assessment (HK-MoCA) in Chinese older adults in Hong Kong. Hong Kong Med J.

[7] Shea YF, Lee MC, Mok MM, Lam MF, Chu LW, Chan FH, Chan TM (2019). Self-care peritoneal dialysis patients with cognitive impairment have a higher risk of peritonitis in the second year. Peritoneal Dial Int.

[8] Bai F, Tan Y, Miao M, Zhai W, Wang Q, Liu L (2016). Correlation of propagated sensation along meridian and TCM constitution. Chin Acupunct Moxibust.

[9] Sang XX, Wang ZX, Liu SY, Wang RL (2018). Relationship Between Traditional Chinese Medicine(TCM)Constitution and TCM syndrome in the diagnosis and treatment of chronic diseases. Chin Med Sci J.

[10] You H, Zhang T, Feng W, Gai Y (2017). Association of TCM body constitution with insulin resistance and risk of diabetes in impaired glucose regulation patients. BMC Complement Altern Med.

[11] Li L, Yao H, Wang J, Li Y, Wang Q (2019). The role of Chinese medicine in health maintenance and disease prevention: application of constitution theory. Am J Chin Med.

[12] Jiang QY, Li J, Zheng L, Wang GH, Wang J (2018). Constitution of traditional Chinese medicine and related factors in women of childbearing age. J Chin Med Assoc.

[13] Wang Q, Bai M, Yang Y, Liang X, Sun P, Han J, Fan T, Yuan CJJ (2018). Application of TCM constitution in lifetime health maintenance. J Tradit Chin Med Sci.

[14] Zeng C, Zhang W, Zhang E, Deng J, Lin M, Zhong M, Li J (2017). Characteristics of TCM constitution in patients with mild cognitive impairment. Acad J Guangzhou Med Univ.

[15] Sun W, Zhang Q, Yang J, Xu J, Lin X, Wei J, Wang R, Zhang X (2018). Investigation and analysis of the distribution of TCM Constitution in patients with mild cognitive impairment. J Xinjiang Med Univ.

[16] Zhang Y, Wang X, Fu Q, Hu H, Wang X (2007). Epidemiological study on TCM syndromes of mild cognitive impairment. J Beijing Univ Tradit Chin Med.

[17] Watson NF, Badr MS, Belenky G, Bliwise DL, Buxton OM, Buysse D, Dinges DF, Gangwisch J, Grandner MA, Kushida C (2015). Joint Consensus Statement of the American Academy of sleep medicine and sleep research society on the recommended amount of sleep for a healthy adult: methodology and discussion. J Clin Sleep Med.

[18] Tuokko H, Frerichs R, Graham J, Rockwood K, Kristjansson B, Fisk J, Bergman H, Kozma A, McDowell I (2003). Five-year follow-up of cognitive impairment with no dementia. Arch Neurol.

[19] Zhan HJ (2005). Social-economic context of parent care: explaining Chinese caregivers' psychological and emotional well-being. J Gerontol Soc Work.

[20] Yu W, Ma M, Chen X, Min J, Li L, Zheng Y, Li Y, Wang J, Wang QJT (2017). Traditional Chinese medicine and constitutional medicine in China, Japan and Korea: a comparative study. Am J Chin Med.

[21] Wang S, Long S, Wu WJ (2018). Application of traditional Chinese medicines as personalized therapy in human cancers. Am J Chin Med.

[22] Wang QJF (2012). Individualized medicine, health medicine, and constitutional theory in Chinese medicine. Front Med.

[23] Wang J, Li Y-S, Wang QJC (2019). Identification of Chinese medicine constitution in public health services. Chin J Integr Med.

[24] Li Y, Li XH, Huang X, Yin L, Guo CX, Liu C, He YM, Liu X, Yuan H (2017). Individualized prevention against hypertension based on Traditional Chinese Medicine Constitution Theory: a large community-based retrospective, STROBE-compliant study among Chinese population. Medicine.

[25] Brownie S (2006). Why are elderly individuals at risk of nutritional deficiency?. Int J Nurs Pract.

[26] Chen Y, Wu Y, Yao H, Luo H, Lin B, Zhang X, Liang X, Sun R, Zhao S, Li Y (2018). miRNA expression profile of saliva in subjects of yang deficiency constitution and yin deficiency constitution. Cell Physiol Biochem.

[27] Hu Q, Yu T, Li J, Yu Q, Zhu L, Gu Y (2019). End-to-End syndrome differentiation of Yin deficiency and Yang deficiency in traditional Chinese medicine. Comput Methods Programs Biomed.

[28] Liu Z, Yang H, Zhang M, Cai J, Huang ZJ, Medicine A (2018). The interaction effect between blood stasis constitution and atherosclerotic factors on cognitive impairment in elderly people. Evid Based Compl Alternat Med.

[29] Zhi-chien H (1993). Principles of diet therapy in ancient Chinese medicine:‘Huang Di Nei Jing’. Asia Pac J Clin Nutr..

[30] Solfrizzi V, Custodero C, Lozupone M, Imbimbo BP, Valiani V, Agosti P, Schilardi A, D'Introno A, La Montagna M, Calvani M (2017). Relationships of dietary patterns, foods, and micro- and macronutrients with Alzheimer's disease and late-life cognitive disorders: a systematic review. J Alzheimer's Disease.

[31] Draper B, Low LF, Brodaty H (2018). Integrated care for adults with dementia and other cognitive disorders. Int Rev Psychiatry.

